# The First No. of the Register

**Published:** 1847-10

**Authors:** 


### *The First No. of the Register.*

The first number of the Dental Register will be sent to those of the profession, whose address we have at hand. Those wishing to get the remaining numbers will please send in their names, with the subscription, at as early a day as possible.

We hope the profession generally will take an interest in sustaining the work. The price at which it is furnished is less than that generally paid for Dental works of about the same size. It is expected that all letters on business to either of the Editors will be postage paid.

The association of Dentists, at whose instance the work is commenced, have undertaken to pay for the First Volume. It is supposed that the funds on hand, and to be paid in by the end of the year, will be sufficient for that purpose. The subscription, therefore, received for the first volume, will stand as a *safety fund* for the expenses of the second, and if, as we hope, some two hundred subscribers can be procured, the Second Volume will either be enlarged or much embellished by engravings of improvements in instruments, anomalous cases in practice, &c., &c. The plan thus adopted secures, at least, the publication of two volumes.

But we look not alone to the Dentists for that wherewith to sustain the Register, for we hope to make it such a work as a large majority of our physicians will commend, and many of them subscribe for, a work, as this is designed to be, more practical than theoretical, should be in the hands of every physician.

It so happens that a great amount of the operations peformed by itinerating dentists, are more or less thrown into their hands by the physicians residing in the towns they visit. How often do we hear such remark—that they have been egregiously deceived in the qualifications of those they supposed every way worthy of confidence. Now a little attention to this matter would enable every physician to determine upon the manner in which an operation is performed. Is not this much due to those who consult them as to the propriety of an operation on their teeth, and who, perhaps, employ the dentist through their recommendation. We know that many take this view of the responsibility, and refuse to recommend any except those whose operations they have seen tested for years; many also procure various works on Dentistry, but most of these are Pathological and do not give direct instruction how you shall determine when an operation is properly performed. We refer particularly to the operations for the preservation of the teeth. Such as plugging, filing, cleaning, &c. We would suppose, indeed, that a practical work of this kind, in which all the operations of Dental Surgery are minutely described, errors exposed, and proper directions given for the preservation of the teeth, would be taken by many families and others who regard their teeth as organs worthy of preservation. It must be remembered that dental operations are required by almost every individual, and that hundreds commence the practice without even attempting to qualify themselves for its duties, regarding preparatory study as so much time lost—but following the maxim that "experience makes perfect," they go to work hoping rather to be paid for their trouble of study, than pay for knowledge honestly acquired. We know many who have paid such much more than two dollars per annum, for years back, that would have saved their money and teeth, had they read half as much dentistry as politics.

---

